# Latino/Hispanic Alzheimer’s caregivers experiencing dementia-related dressing issues: corroboration of the Preservation of Self model and reactions to a “smart dresser” computer-based dressing aid

**DOI:** 10.1177/2055207616677129

**Published:** 2016-11-21

**Authors:** Diane Feeney Mahoney, David W Coon, Cecil Lozano

**Affiliations:** 1 School of Nursing, MGH Institute of Health Professions, Boston, MA, USA; 2 College of Nursing & Health Innovation, Arizona State University, Phoenix, AZ, USA; 3 School of Computing, Informatics and Decision Systems Engineering, Arizona State University, Tempe, AZ, USA

**Keywords:** Dressing, Latino or Hispanic caregiver, context-aware computing, qualitative research, gerontechnology, smart home, assistive technology, dementia, Alzheimer’s disease, cross-cultural study

## Abstract

**Objective:**

To gain an understanding of Latino/Hispanic caregivers’ dementia-related dressing issues, their impressions of using a “smart” context-aware dresser to coach dressing, and recommendations to improve its acceptability.

**Method:**

The same Latina moderator conducted all the caregiver focus groups. She followed a semi-structured interview guide that was previously used with White and African American family caregivers who experienced Alzheimer’s disease related dressing challenges. From that study, the Preservation of Self model emerged. Using a deductive qualitative analytic approach, we applied the thematic domains from the Preservation of Self model to ascertain relevance to Latino/Hispanic caregivers.

**Results:**

Twenty Latino/Hispanic experienced caregivers were recruited, enrolled, and participated in one of three focus groups. The majority were female (75%) and either the spouse (25%) or adult child (35%). Striking similarities occurred with the dressing challenges and alignment with the Preservation of Self model. Ethnic differences arose in concerns over assimilation weakening the Latino culture of family caregiving. Regional clothing preferences were noted. Technology improvement recommendations for our system, called DRESS, included developing bilingual prompting dialogs and video modules using the local vernacular to improve cultural sensitivity. Caregivers identified the potential for the technology to enable user privacy, empowerment, and exercise as well as offering respite time for themselves.

**Conclusion:**

Findings suggest dementia-related dressing issues were shared in common by different racial/ethnic groups but the response to them was influenced by cultural dynamics. For the first time Latino/Hispanic voices are heard to reflect their positive technology impressions, concerns, and recommendations in order to begin to address the cultural digital disparities divide.

## Introduction

In 2015, 15.7 million family and friends provided 17 billion unpaid hours of dementia caregiving valued at more than $217 billion.^1^ As the US population ages, the Alzheimer’s Association (2015) predicts the number of people with Alzheimer’s disease (AD) will increase from the present one new case every 67 seconds to one case every 33 seconds by 2050.^1^ As the disease advances, it becomes more challenging for those afflicted to perform activities of daily living (ADL), thereby heightening the need for practical ways for families to assist them.

Family caregivers identify dressing as one of the most pressing daily needs.^2^ Although dressing tips are publically available on the Internet and in support groups, caregivers often use trial and error methods for 5–6 years before seeking this information.^3^ Moreover, evidence-based interventions to assist caregivers are absent in the literature. Studies on medical textiles and disability wear have focused more on the stigmatizing aspects of patient wear,^4^ and have not attended to less cognitively taxing dressing approaches. People with dementia (PWD) become unable to choose appropriate clothing, organize, and sequence self-dressing. Prior research with White and African American family caregivers found that they provided frequent coaching to preserve the PWD’s sense of autonomy and dignity. Over time caregivers became frustrated by repetitive prompting and supported the idea of using a “smart dresser” to automate verbal and pictorial cues.^5^ Notably missing in both the dressing and technology literature is Latino/Hispanic representation, including those over age 65 who are projected to be the largest ethnic/racial minority in this age group by 2019.^6^

Given the limitations in the literature, our first aim was to gain an understanding of Latino/Hispanic caregivers’ dementia-related dressing issues and to assess whether there were any culture-related aspects. The second aim was to obtain caregivers’ impressions of our prototype smart dresser system, being designed to guide PWD in dressing, and to obtain their suggestions for improvements.

### Conceptual framework

**Figure 1.**
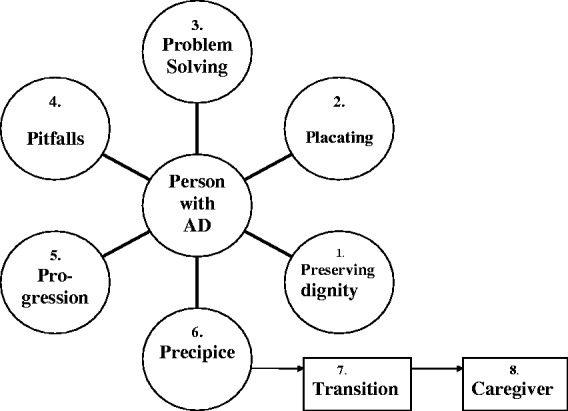
Preservation of Self model (© 2013 Mahoney, all rights reserved).

The Preservation of Self model: care recipient to caregiver emerged from a prior inductive qualitative grounded theory study conducted with White and African American family caregivers caring for PWD who had difficulty dressing wherein data trustworthiness and credibility checks are reported.^3^ The model comprises eight thematic domains, representing stages of caregiving from early through the advanced stages of Alzheimer’s disease: (1) maintaining PWD’s dignity, (2) placating, (3) problem solving, (4) facing pitfalls, (5) unpredictability, (6) precipice point, (7) transition time, and (8) self-preservation (see Figure 1).

The model posits that caregivers initially attempt to preserve PWD’s dignity by taking the blame for their dementia-related dressing problems. Over time, difficulties escalate and caregivers try problem-based strategies that have meaning to the person to respectfully sustain their dressing involvement, even when physical assistance is necessary. Finally, when caregivers confront serious threats to their physical or emotional well-being due to caregiving requirements, the focus shifts from the PWD to preserving the caregiver’s self. This model provided the framework and underlying constructs and code categories for the present inquiry to enable an understanding of the similarities and differences in issues and perspectives by Latino/Hispanic caregivers. Prior to commencement of the study, the research protocol received Institutional Review Board approval from Arizona State University as an exempt study.

## Method

Project outreach staff and community liaisons (*promotores*) recruited participants from summer through the fall of 2014 through personal contacts, support groups, fliers at churches and senior centers serving the Latino community in a large urban southwest area of the US. To be eligible to participate, respondents had to be a Latino/Hispanic family member or friend who directly helped a person, diagnosed with late early to middle stage Alzheimer’s disease, get dressed for at least 5 days a week over a six-month period. In addition, participants had to be able to converse in Spanish (bilingual participants were welcomed), have the ability to hear and speak in a group setting, and give verbal and written informed consent. Caregivers without dressing experience were excluded to ensure information rich participants. Telephone screenings determined final eligibility and gathered additional characteristics of the caregivers and their care recipients. Our sampling goal was not to attain a specific number, but to purposefully engage Latino/Hispanic caregivers with extensive dressing experiences to foster productive discussions that ultimately result in data saturation.^7^ Participants were recruited to include diversity in gender, role, age, and living arrangements.

### Focus group procedures

The number of participants per focus group was designed to be between 5 and 10, large enough to encourage discussion and small enough to facilitate everybody’s participation.^8^ Sessions commenced after the moderator and *promotora* helper discussed and obtained signed consent forms. All forms and handouts were available in both Spanish and English. The same bilingual Latina PhD-level moderator (CL) facilitated all of the focus groups in Spanish (participants’ preference), using the same semi-structured interview guide and funnel technique (global open-ended to focused questions) previously used with White and African American Alzheimer’s family caregivers to ensure obtaining comparative data.^9^ Each session lasted approximately 2.5 hours and consisted of the following phases: (1) moderator’s review of session purpose, confidentiality assurances, choice of pseudonyms, invitation for positive and negative input (10 minutes); (2) participants’ discussion of general dementia-related dressing issues interspersed with moderator’s probes to assess, confirm, validate, refute, and elaborate upon thematic issues (one hour, then 10 minute break);^10^ (3) moderator’s Spanish narration of a 5-minute demonstration video showing the prototype dresser’s components, installation, and proposed in-home operation. Given the high cost and complexity of initial prototyping, only one non-mobile dresser system could be built. Consequently, to accommodate the focus groups in three geographically different locales, we developed the video showing an older male actor portraying a PWD using the prototype. The moderator prompted the participants to discuss acceptability, applicability and usability of the system in general and for each of its features. They were strongly encouraged to share not only positive, but also negative opinions; and (4) session recap to summarize and validate interpretations with the participants, also known as member checking, to ensure the trustworthiness of the data. Upon conclusion, participants received a $40 honorarium and local caregiving resource information.

### The technology

**Figure 2.**
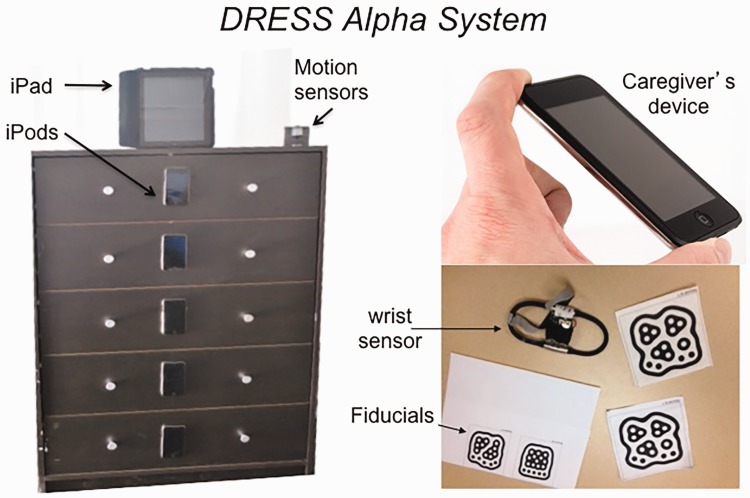
Handout displaying DRESS system components (© 2014 Mahoney & Burleson, all rights reserved).

**Figure 3.**
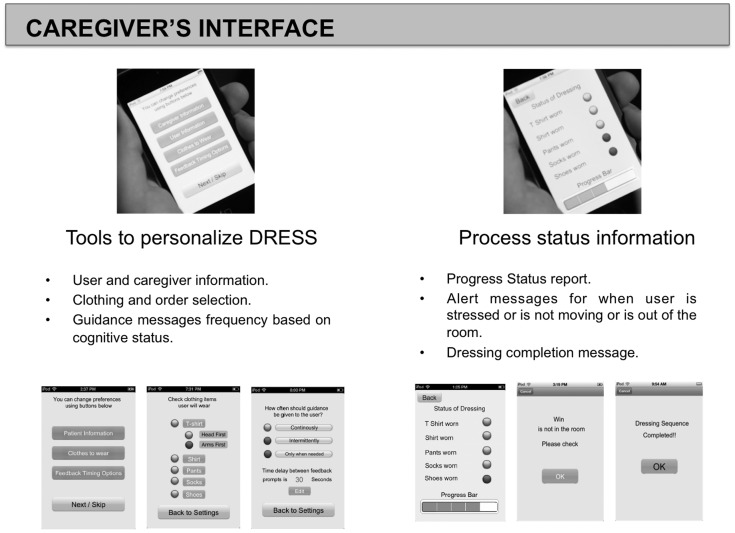
Handout displaying sample IPod screens (© 2015 Mahoney & Burleson, all rights reserved).

Technical details of the system plans, which have been published previously,^5^are updated here. The system is being designed as a cognitive assistive device for PWD who have difficulty organizing, sequencing, and completing dressing by themselves. Based on recommendations from the prior non-Latino focus groups, we revised our design to now offer personalized verbal and video cues through an iPad (to mimic a TV) placed on top of the user’s bureau dresser. A computer base station, hidden in or under the dresser, receives inputs from an array of drawer and room sensors, clothing sensor tags, and a privacy camera that can capture body outlines and movement without revealing personal body parts. The PWD will wear a watch with an imbedded wrist sensor that transmits his/her skin conductance response state to the computer. Custom-designed algorithms parse the data and adjust the type of dressing task guidance, based upon actual conditions, and delivers prompting cues tailored to the user’s situation through the iPad. Potentially caregivers will be able to do other activities or gain respite time until the system notifies them, via a text message that either help is needed or dressing is completed. The elements of the system (Figure 2) and the caregivers’ device interface screen (Figure 3) were verbally described to the participants accompanied by handouts in both Spanish and English.

### Analysis

We employed a deductive grounded qualitative method to address the study aims. Deductive inquiry starts with an established model, and seeks to ascertain the fit, in different instances.^11^ In the present study, we explored the fit of the Preservation of Self model to different participants, namely Latino/Hispanic caregivers, by replicating the focus group study protocol for data collection and applying the code categories derived from the model. Our analytic approach followed pattern matching using Spradley’s principles for qualitative comparisons:^12^ (a) the similarity principle determines categories by looking for units of information with similar content to the model’s eight domains through constant comparative analysis, (b) the contrast principle directs the search for mutual exclusivity, or exceptions to the study model.

The focus groups were audio recorded and transcribed in Spanish then back translated into English by the moderator (CL). The transcriptions were read line by line by two analysts (DFM & DC) first independently and then together through peer debriefing to clarify the meaning of the raw data and to ensure mutual agreement on the translation. Using an iterative analytic process, data were re-reviewed to code descriptors, categories, and patterns with mapping to the eight themes. If any discrepancies occurred, the researchers discussed them until they reached 100% consensus. In stage 1, we coded the open-ended and semi-structured interview data according to the Preservation of Self model’s domains, and assessed congruent (similar) and non-congruent (different or divergent) constructs or themes. Then, in stage 2, we explored differences related to cultural aspects and regional location. Finally, in stage 3, we conducted an analysis of the caregivers’ impressions of the proposed smart dresser. Data analyses involved question-by-question and line-by-line continual comparing and contrasting of meanings until pattern and data saturation was achieved, signaling completion.^8^ Findings were shared with the moderator and *promotores* for structural and interpretive corroboration. The results are presented with thick descriptions supporting interpretive validity and to allow readers to assess the transferability of the findings to their contexts.^13^

## Results

### Participants

Twenty caregivers (4 male and 16 female) all self-identified as Latino or Hispanic were recruited, enrolled, and participated in the focus groups. They were predominantly in their early sixties but ranged from 32 to 80 years of age. The participants were spouses (*n* = 5), adult children or children-in-law (*n* = 7), a sister (*n* = 1), adult grandchildren (*n* = 3), and paid helper/friends (*n* = 4). Almost half lived with their care recipients who were mostly in the middle stage of Alzheimer’s disease. On average, the participants have been caregiving for almost five years (M = 4.78 years; SD = 1.16), stating that they were either the only person who provided any substantial amount of care (20%), they provided most of the care (50%), or they shared care responsibilities about equally with others (30%). Their caregiving involvement was reflected in the high average number of hours per day they estimated they were actually “doing things” (M = 14.50; SD = 9.02) or feeling they needed to “be there” or “on duty” (M = 22.10; SD = 4.66) to provide help for their care recipients. The caregivers participated in one of the three focus groups held across the region, which resulted in 9, 6, and 5 session members, respectively.

### Thematic similarities – Preservation of Self model congruence

The discussions that emerged from the members were first mapped to the constructs and existing codes for the Preservation of Self model following the deductive approach previously described.^11^ Numerous instances of text aligned with the model’s codes and constructs. The following section presents sample exemplars to support the model’s fit. Please note the names are pseudonyms, followed by the number of the focus group and transcript page(s) that show the links to the audit trail in support of data credibility.

1. *Preserving personal dignity.* Latino caregivers readily related the importance of respecting their loved ones by and in the way they dress. Dressing was an integral part of who PWD once were and now it is becoming a stumbling block, a daily reminder of decline, and a task to be increasingly negotiated in a thoughtful manner. Barbara 2/5: “She [mother] has also buttoned the blouse crooked … she begins to button, and I tell her in a way not to hurt her.”

Canción 1/6 &11: “He very much liked to look handsome, [with] his tie, hat and everything. But the difference we noticed … if he spilled food on himself we wanted [him] to change and he would say why? [But if his shirt] was buttoned wrong and we would tell him: Look; and he would say: leave me alone … you hesitate a little out of the respect that you have for them but at the same time you want to help them because they can’t do it.”

2.* Placating.* Many of the caregivers sought to maintain the peace and avoid confrontations over dressing issues by removing incorrect clothing choices and minimizing, not emphasizing, any errors resulting from PWD’s efforts.

Jessica 2/5: “My husband … was putting back on the clothes that he had taken off … he couldn’t tell the clean clothes from the dirty ones. So I would say, “this one you just took off.’ And he would say, “No, no that can’t be” … So I started hiding his dirty clothes… And he would button [his shirt] all wrong. But I wouldn’t tell him you’re putting it on wrong, I would just tell him it looks a little different and I would fix it.”

3.* Problem solving.* Over time, caregivers seem to develop a repertoire of useful strategies, ranging from avoidance of known difficulties to motivational approaches that align dressing with favorite activities.

Blanca 1/12: “The lady I took care of loved to watch John Wayne movies a lot. She would make me take out all of her blouses from the closet and say ‘no, not that one,’ ‘no, that one.’ I would take it off the hanger and put it on her [and she would say], ‘no, not this one either,’ ‘no, not that one.’ [I could get her to stop by saying,] ‘Come on, now get dressed because we are going to watch a John Wayne movie.’ She would say ‘Oh, okay, okay. Yes, yes!’ She never forgot him you know … She would forget to eat, she would forget to go to the bathroom, and she would forget everything, except John Wayne!”

4.* Pitfalls.* Eventually, the strategies became less successful and problematic behaviors escalated into non-productive situations that were frustrating for both the caregiver and the PWD. In this example, bathing triggered the resistance to dressing.

Lily 1/8: “When I take her out of the tub, she gets out of the bathroom and doesn’t let me dress her. [I say] ‘Come on, let’s get you dressed,’ and she says, ‘no, I don’t want to,’ and there I am with the clothes chasing her all over the house [with her yelling,] ‘No, I don’t want to!’ She has a little chair where she goes and gets into a ball.”

5.* Progression.* Participants realized that marked personality changes indicated a worsening of the disease and led to more challenging caregiving. Family members experienced great emotional strain when they were yelled at and told to go away.

Marta 2/13: “If she [aunt] was in a difficult moment, helping her was going to be hard … it was hardest when she was screaming, ‘Go! I don’t want to see you!’ to not feel bad and take it personally.”

6.* Precipice.* Eventually the demands of the caregiving situation exceeded the capabilities of the caregiver(s). This was more noticeable with spousal or older adult caregivers who were frail and had their own health problems that adversely affected their caregiving abilities.

Lily 3/9: “My father had someone placed to care for him and didn’t want the help. He only wanted my mother, but now she uses a walker and can’t do his care. We would tell him, ‘Here is the person to help you’, and he would say, ‘NO.’ It was very difficult.”

7.* Transition.* Families frequently turned to external helpers to buttress their caregiving and protect the older vulnerable caregivers. They would employ non-family caregivers who could provide the needed services.

Bárbara 2/15: “My mother would get tired. She would stress. When you are tired, you get impatient, and then he [grandfather] doesn’t want to do things. … So we placed a helper for my mother … half a day for the lady to care for him, and the other half of the day, my mother would care for him.”

8.* Preservation of the self.* By the last stage of the model, attention fully shifts from preserving the self of PWD to that of preserving the well being of the family caregiver(s). Competing demands for their own personal health and welfare overcome their capacity to provide the intensity of needed caregiving. For these caregivers, it meant they sought more full time help, usually trusted Latino friends and acquaintances that were known to be warm and loving.

Blanca 1/2: “I began cleaning their house and we (the family member and her) began to develop trust and she considered me her friend and I did her, and when I saw she was having problems with her mother I offered [to help] … she didn’t have anybody to leave her with … I offered and I cared for her. I took care of her with lots of love because she was the mother of my friend.”

### Phase 2: cultural differences

The second phase of the study searched for any views or themes that deviated from the model, and found no disagreement with the key components. As expected, cultural differences did emerge related to the participants’ Mexican heritage and Christianity’s influence. Latino/Hispanic caregivers frequently attributed caregiving abilities to God’s enablement. God was seen as underpinning the caregiving situation and engendering the physical and emotional capacity to do the necessary caregiving. Jessica 2/2: “It is difficult … [but] God has given me patience and the love to care for my husband.”

Several participants mentioned that they viewed the responsibility for elder family caregiving as part of their cultural heritage. One participant conveyed a long-term family commitment.

Barbara 2/15: In Mexico, my family didn’t have resources. There is you and your family, nothing more. … I’m taking care of my mom now. We had previously cared for my grandfather until he died at 105 years old. We were able to take care of him: to feed, dress, bathe, clean his ears, cut his nails and so on. Right now we are starting up with my mom. We’ve been at it around 4 years and she’s 77 years of age right now … we gladly do it. I think that it’s part of our Mexican-American culture to help our elders.”

*Regional dressing preferences.* Wives reported their husbands had a strong affinity for their Western boots, ties, buttoned down shirts, pants and belts, despite their lack of cognitive ability to perform the coordinated maneuvers necessary to apply them. Substituting elasticized sweat suits for these men, while easier to put on, was not deemed acceptable by them for their age and gender in this Southwest region.

Rosa Esther 3/17: “He sees his clothes hanging in the closet, his shirts, his pants, everything he would wear including his boots; everything he wants to wear like before and he can’t. It’s a problem. I would like him to be able to dress like before and wear the clothes he has, but I can’t let him. If I have so much difficulty helping him put on clothes that are easy with elastic and all, how am I going to be able to get his other clothes on him? It’s very difficult … he can’t use a zipper, he can’t button his shirts or pants … He blames me … I have him dressed in women’s clothing!”

*Changing caregiving values and response to technology.* Participants expressed different perspectives on the cultural expectations for family caregiving and the use of technology. The dominant view supported a strong cultural preference for caring for one’s elders by the family. Ester 2/22 daughter: “This would work for families who have no other option but to leave their older relatives alone. But, typically the Mexican culture is to be cared for by the family.” Several participants, predominantly the paid helpers, noted that not all families are currently able to provide caregiving. Mike 1/17: “Lots of people that have dementia don’t have any family here; the family doesn’t want to bother with them. The caretaker is someone they don’t know (paid helper).”

Technology usage emerged as a potential aid, especially for the next generation of elders, the baby boomers that are using computers to maintain long-distance family relationships and are open to new advances.

Marta 2/21: “With my grandmother, the culture and all of that … the technology was not something she used, but my mom now, she knows … at her job she uses the computer, email. She learned a lot and if something happens to her or to my dad, this technology would be more acceptable and if it happens that they get Alzheimer’s, this would help them. … My dad, he is a very private person, [and wouldn’t want us] to see him. [naked]”

### Phase 3: impressions of the assistive dressing technology

We wanted to learn if the technology system design using an ordinary home, dresser would integrate into caregivers’ routines and home environment and found general agreement and support for this mode.

Lily 3/35: “Looking at that chest of drawers I think that it would function because I am remembering how my father would get in front and open his drawers … I would ask, ‘What are you looking for?’ He would say, ‘I don’t know.’ He would open the drawers, he didn’t know for what, but he would open them. If in that moment you tell him, ‘take out the socks and put them on,’ then he doesn’t have to think … with such direction he could do it.”

*Respite*. Participants were queried about whether they might use the 30–45 minute dressing session as a “respite break,” that is, to do something more restful while the system oversees the dressing process. Prior research has found that this short interlude, when used to do something perceived as pleasant by the caregiver, helped to reduce caregiver stress.^14^ Most participants agreed that they’d welcome a break to rest or complete other tasks. Bárbara 2/31: “For me, the benefit is being able to rest. Having something the person can do for themselves with just a little bit of help from technology, well, that would give me a little time to rest, or to do other things I have to do.” Jessica 2/31: “I also think that the benefit is not only to dress but the sensor also helps because they won’t get out without you knowing and that helps a lot. Because one gets very tired, you dose off and fall into a deep sleep … it will alert me if he wanders out of the room.”

#### Technology benefits

Caregivers were then asked an open-ended question to query the possible benefits this technology might offer them. They identified the following (see Table 1):

**Table 1.** Hispanic/Latino perceptions of benefits and barriers to technology usage.

Benefits	Barriers
Validates memory loss	Wrong stage of AD – too early or late
Empowers user	Users’ visual and sensory perception problems
Always talks in a calm voice	Requires a change in routine
Mental and physical exercise	Caregivers’ need to “be there”
Promotes privacy and dignity	Instability of new technologies
Caregiver respite/wander alert	Affordability of technology

*Validates memory loss.* Caregivers saw the technology’s potential to provide a critical source of information to inform family members who did not recognize or denied their relative’s memory loss. They felt recording the time to complete dressing, how many prompts, and the number of dressing errors as useful measures to help recognize cognitive and functional declines over time.

Marta 2/19 & 20: “In the beginning, there were [family] arguments that were like fights. I think a system like this would have helped convince those who didn’t believe us. ‘Look, it is noticeable that she is not remembering, she is not functioning.’ And it would have helped us to talk with and show the doctor.”

*Empowers user.* Participants liked the way the system adjusted to the person’s situation and encouraged self-dressing. They felt as if they had taken away dressing independence out of necessity and now might be able to return some, if not all, independence in dressing. Laura 2/18: “He would feel more comfortable this way and feel useful instead of being handed everything (by wife).”

*Always talks in a calm voice.* Some caregivers reported interpersonal difficulties when trying to repeatedly guide dressing routines. Most had noticed increasing tensions due to resistance over dressing, frequently resulting in louder tones and more angry voices that only worsened the situation. They valued DRESS’s persistent neutral calm voice. Lily 3/36 & 37: “I saw it with my father, if my mother gave him an order he did not want to follow it … I believe it is very important that it be a calm voice that is nice.” Sherlyn 3/36&37: “A soft loving voice … if a man’s, not macho.”

*Mental and physical exercise.* Participants identified an activity benefit from encouraging PWD to dress themselves by exercising not only their minds, but also their bodies.

Blanca 1/13 & 14: “When they have Alzheimer’s, they are definitely sitting doing nothing, and the only thing they are doing is stiffening their muscles. I think that’s [DRESS system] perfect, because it helps to exercise their hands, their legs, their mind.”

*Promotes privacy and dignity.* The caregivers did not view the technology as violating their privacy because they viewed it as offering a new means to help maintain or achieve privacy for the PWD during dressing. Many participants noted not only they were uncomfortable being in the presence of an elder while they undressed but also they felt it diminished their dignity.

Marta 2/23: “I am seeing that the sensor would let us know [dressing status] even though we close the door. She will be able to get dressed alone … that is what she likes, to be private. The same with my grandfather, [he would yell] ‘I want to be alone to dress’.”

### Barriers to technology usage

Equal time and emphasis was given to querying for negative concerns about the technology and the discussion revealed the following:

*Wrong stage of AD.* Several recognized that their PWD, now in the advanced stages, would not be able to use this device and affirmed our plans to target people in late early to early middle stages.

Sherlyn 3/45: “I think that that system is for a person who’s in an intermediate stage; someone whose illness has already begun and is now progressing, but not for someone whose disease has already become very advanced … if they have it in front of them they won’t know what to do even though it tells them.”

*Visual and sensory perception problems.* Others raised concerns that their PWD’s existing problems with understanding sensory inputs might affect their usage and suggested accommodations. One had a care recipient that experienced TV induced hallucinations and was concerned that the iPad screen “talking” would create a similar situation.

Estrella 3/36: “My mother has very poor vision so the size for her to be able to see the pictures or letters has to be larger. When the television is on, she isn’t looking at it, but she says, ‘See there that lady?’ … I just realized that the people that she was telling me about were in the news. So I wonder if she sees a device like that [will it cause similar confusion?].”

*Changes the routine.* Introducing new routines to PWD is challenging and often evokes resistance. Caregivers voiced the need for motivational encouragement and reinforcement until it becomes an established routine.

Estrella 3/31: “It could work if I explain it to her and tell her, ‘Look, we are going to try this. We’re going to play. Let’s see what you think of the game.’ … She might accept it today, but tomorrow she won’t want to. And I convince [her again] in the next week and again, it might function in that way.”

*Caregivers’ need to “be there.”* Caregivers wrestled with the desire versus the need to be there with and for the PWD. Some believed the person still made a connection and knew at some level the caregiver was there and loved them. Other participants relinquished being there to trusted friends to ensure oversight safety. Julia 1/14: “Personally, I think it is very important to make them feel that we are there with them, but there are moments they may get mad with us and say ‘don’t help me,’ but even with that anger, I say they want us.”

Canción 1/14: “We looked for a person when I couldn’t be there all the time, but she would say, ‘no, no, I don’t want that’. She would only use the person to talk with, not to do things for her. But she [helper] is there to watch over her, to see what is going on.”

*Computer instability and lack of reliability.* One participant did not see the possibility of giving up supervising dressing to a computer due to technology’s lack of reliability and vulnerability to network and performance problems. Julia 1/17: “Maybe it would save some time for a caregiver, but if something happens, you can’t be doing other things; computers fail and one has to always be alert.”

*Affordability of technology.* How to offset the high cost of technology for lower income people was prominent in the discussion. Participants suggested several ways to make future access to this type of technology more affordable should it be commercialized. Martha 2/32: “I imagine the person will get to a point they will advance or something will happen physically, and the [end] time has come. Let’s say it cost two thousand dollars. I return it and there’s a credit.” Jessica 2/32: “It would be easier to rent because the time will come when you will no longer use it. So another person can rent it again.”

#### Device features and recommended modifications

Participants were queried about the smart dresser features to gain their impressions, cultural relevance, and solicit recommendations.

*Dresser.* First we asked about their usual choice of clothing storage to see if it would be a closet, wardrobe, or anything other than our plans for a dresser bureau with drawers. Chest of drawers was the local term and the use of drawers was universally accepted. Blanca 1/23: “Chests of drawers have always been used … since we were children”. We planned to integrate personalized music into the coaching to divert agitation and the participants supported this idea if the music was familiar to them. Jessica 2/24&25: “But don’t put on loud music, [use] something that will relax them … music that is most pleasing to them.” We asked about mounting a mirror on the side of the dresser to allow those who can use it to check their appearance, and everyone agreed this would be helpful. Estrella 2/28: “My mother likes to have the mirror there because she still recognizes herself.” Adding a chair next to the dresser was recommended in all the groups for comfort and balance safety. Estrella 3/42: “They can lose their balance when they are not sitting down.”

*Fiducials.* Fiducials, clothing tags similar to a barcode that allow the camera to detect clothing orientation without seeing body parts, received mixed reactions. Two caregivers thought their person with dementia would not notice or remember the tags while others recommended blending them within clothing designs to disguise them. Jessica 2/26: “Well, I think we would use it [tags] in the house, but not to go out. Maybe they won’t notice them so much, but we would.”

*Dialogs.* From watching the video simulation of the dressing coaching, participants recommended more personalized approaches to the audio dialogs by using culturally familiar endearments and nicknames and possibly their own voices and pictures. Ester 2/24: “A very cordial message, ‘Mami, it’s time to get up and get ready, open the top drawer’ … Try to calm her down (calm soothing voice), ‘Mami, don’t worry, that is not the drawer, but don’t worry, look, let’s start over again. Let’s open the top drawer first and there is your blouse,’ like that.” Bárbara 2/21: “I think it should be the voice of the person that is caring for them so she would feel more familiar and would think the person is there.” Others, who experienced fighting over dressing, disagreed, preferring a new “neutral” voice.

*Run-in adjustment period.* Three participants brought up the need to test the system’s operation and capability to tailor to the PWD’s needs. One recommended that we have a planned run-in period to test operations and tweak the settings to optimize customization.

Martha 2/30: “have a period like a week, then adjust the system because it would function perfectly, let’s say in Jessica’s house, using everything. Then take out some things, because that is how it would work better for Barbara in her house … Accept it like it is (with all the features), install, then adjust it.”

*Affirming.* Overall, the vast majority of the participants (95%) supported the effort of designing new technologies for caregiving in general, and this intervention in particular. Blanca 1/24: “Well, that it is perfect and it makes me happy that they continue to come up with more, more help for all the persons that need it … you guys are going through the work to come up with all of this, it is very good.” Paloma 3/47: “I hope that there will be things like this, if one day I get dementia.”

## Discussion

Notably, Latino/Hispanic caregivers reported similar dementia-related dressing issues to those previously reported by White/African American caregivers. The behavioral manifestations of middle stage Alzheimer’s disease related dressing issues appears similar across cultural groups and aligned with the constructs and themes from the Preservation of Self model without any major deviances. For example, Latino caregivers similarly tried to offset the care recipients’ difficulties by taking the blame for missteps and using a variety of strategies to encourage dressing. Knowledge about caregiving also primarily came from family, friends, and through trial and error. Transcripts revealed that many family members used patterns of communication that made unrealistic demands on PWD, and resulted in bilateral frustrations. Prior research has indicated that non-Latino PWD and their caregivers are infrequently seen by clinicians in the early disease stages and that families attempt to negotiate behavioral changes and difficult interactions without professional guidance.^3,15^ We found similar findings in this study and recommend more outreach and earlier caregiver referrals to local resources for more proactive caregiver dementia skill building and support.

Cultural differences influenced the response to needing help with dressing. *Familismo,* or being cared for by family members, has been a strong tradition in Latino families.^16^ We found that some older family members worried that this tradition was being weakened by the “American” lifestyle that they see as devaluing family caregiving. Gelman’s recent in-depth analysis of *familismo* reported discrepant views on its current cultural relevance. Some family members believed it facilitated caregiving by reinforcing traditional expectations of helping, while others disavowed its’ contemporary relevance.^17^ Others report that baby boomer caregivers appear more open to outside service supports to succeed in juggling caregiving, work, family, and social commitments.^18^ We did find some Latino/Hispanic caregivers were open to employing lay friends or helpers to provide care. Four participants, who were paid helpers, reiterated a theme of “love” for older people and a need by the family as the motivation to trade other similar paying employment for this more personally gratifying position. They also mentioned being “trusted” by the families, coming from within the same community, and being known to them, as important factors. When queried, however, they did not relate any concerns about use of this technology eroding their employment opportunities.

From the technology perspective, a surprising finding was the participants’ level of enthusiasm, in response to this technology in particular, and the potential for caregiving technologies in general. All participants agreed with the intent to develop and use DRESS with PWD, but they did emphasize matching it to the right stage of dementia and investigating its’ limits. One participant questioned whether a voice coming from the dresser would cause hallucinations. Concerns about inducing hallucinations, from using interactive prompting technologies, have been raised by critics, but they have not occurred in the auditory coaching research reported to date.^19,20^ We will, however, specifically observe for any new onset or exacerbation of hallucinatory experience when the system is tested with PWD.

Contrary to expectations of technology critics, the participants viewed this technology as a means to provide privacy by enabling the PWD to dress without the intrusiveness of a caregiver being present. In addition, prior research has shown that it is very stressful for adult-children to dress a parent, especially of the opposite gender.^3^ A recent qualitative study of eight Mexican-American sons who are providing personal care to their mothers offers further insights into this emerging phenomenon.^21^ Having an automated dressing aid was viewed as a buffer; reducing the intrusiveness and intimacy related stressors inherent with hands on dressing assistance. Caregivers also reported difficulties with family members and doctors who did not believe them when they reported cognitive concerns. They saw the technology’s event record as a means to provide objective documentation to measure and track the decline in dressing abilities. They hoped this type of information would be useful to gain family and provider recognition of the seriousness of the memory loss and impairment of self-care abilities. They also endorsed the cognitive stimulation and physical activity the system offered the PWD, as a means to “exercise their minds” and move their bodies to dress. Rather than passively sitting and having things done to or for them, the caregivers saw the technology as offering a new way to engage PWD.

The study findings did reveal the need for the technology to display more culturally sensitive training and prompting video-clips. The current video, featuring a white male dyad, was seen as being too “cold,” with need for more affection and familiar endearments. Caregivers mentioned that they typically embrace and are more demonstrative and asked if we could use additional words/pictures/songs to display more “warmth.” As a result, we see the need to develop a new version, with video clips and promptings in Spanish, with a Latino/Hispanic dyad using the recommended interaction style.

We also found that the clothing categories included in our system narratives needed to be expanded given the appeal of “Western wear,” especially for the Latino men in this region. One husband resisted wearing easier to put on sweatpants because he insisted that “men only wear pants with belts.” As a consequence, we will alter our scripting to include narrative options for Southwest regional style rope ties, boots, and belts and consider other meaningful items. We will approach these alterations as options and not stereotypes by constructing alternative scripts that are congruent with the model’s theme of preserving personal dignity, whether that sense of personhood is exemplified through the PWD workplace “uniforms” or their preferred home or leisure time apparel. Participants suggested integrating the fiducial tags into the clothing designs to make them less noticeable. Currently for prototyping, the fiducials offer the most cost effective and reliable way to provide system testing. We are monitoring the rapidly developing market for sensor imbedded fabric and plan to integrate that technology when it becomes economically feasible.

**Figure 4.**
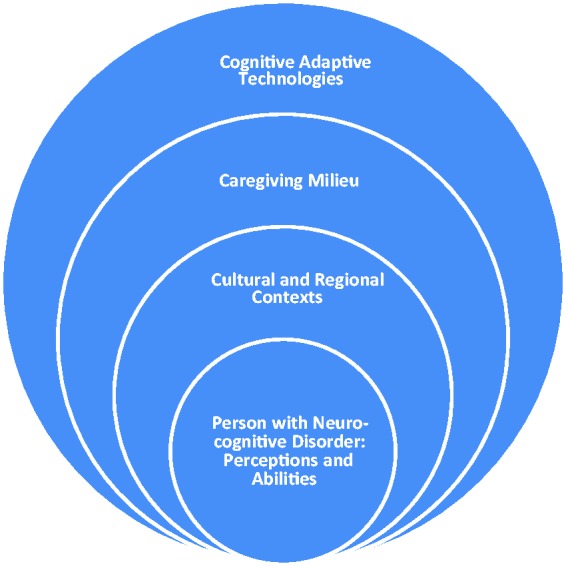
Person-centered Model for Cognitive Adaptive Intervention Technologies.

The Latino caregivers in this study identified that the DRESS technology offered a new way to make the lives of those they cared for better by promoting autonomy, exercise, and dignity, all aspects called for in developing interventions for older adults.^22^ Although person-centered care has been mandated by federal legislation, substantial barriers exist that include negative beliefs about its feasibility for people with cognitive impairment given co-morbidity, culture, race, ethnicity, language, and socio-economic challenges.^23^ It is important to note that the Latino caregivers viewed this technology intervention as a respectful way to help PWD by taking into consideration their individual abilities, needs, and cultural preferences. Given our present “Internet of Things,” comprised of 25 billion electronic devices connected to the Internet this year and 50 billion of these devices forecasted by 2020, along with the emerging “Internet of Things” business models, there is a tremendous opportunity for person-centered innovations in technology.^24^ Emanating from this research is a model that portrays that perspective to guide future developers. It features the PWD at the center surrounded by their cultural and caregiving milieu. Technology appears in the outmost circle, influenced by and needing to be responsive to the inner realms (see Figure 4).

### Limitations

We were mindful that “structured focus groups run the risk of limiting the discussion to the topics the researchers want to hear about rather than revealing the participants’ own perspectives.”^8^ In response, we did employ a Latina cultural insider as moderator to enhance trust, and she not only followed the semi-structured interview guidelines, but also offered participants multiple opportunities to add their own perspectives. Generalizability does not conceptually fit with small samples chosen specifically for their homogeneous characteristics typical of qualitative research.^25^ Thus we do not suggest extrapolation of our findings beyond our sample, but offer numerous illustrative quotations to enable readers to determine the dependability and transferability of findings to their settings. From the technology perspective, given the initial stage of technology development and budget constraints, we did not have three fully developed systems for the focus group demonstrations. Instead we showed a video of the prototype mock up version with handouts showing pictures of the components. The caregivers, however, did not have any apparent difficulty perceiving the system design, features, and intention.

## Conclusions

We engaged Latino/Hispanic caregivers as partners in our smart dresser system development. We found that they had predominantly similar dementia-related dressing issues to previously reported research with White and African American caregivers, but several differences due to cultural perspectives, linguistics, and geographic location. Both the similarities and differences have important implications for future technology iterations as we aim to address the needs and desires of as many diverse caregivers as possible. Our participants viewed DRESS as a new and needed means to record memory loss and its’ progression, provide mental stimulation, physical exercise, and privacy in dressing, using an aide that always has a calm voice and never gets upset when repeatedly prompting the PWD. These are fertile areas for hypothesis generation. Future research is needed to ascertain whether the caregivers’ positive attitudes towards this technology translate into technology adoption and improved outcomes when DRESS is ultimately tested as an in-home intervention for with PWD and their caregivers. Meanwhile, the participants’ enthusiasm for embracing new technologies to assist with caregiving should encourage other technology developers to pursue other innovative offerings, mindful of end-users’ wants and in alignment with our Person-centered Model for Cognitive Adaptive Intervention Techologies©. In summary, findings from this study uniquely document the impressions and input of Latino/Hispanic caregiving participants, a greatly under-represented minority group in the technology intervention development literature, and contribute to bridging the digital disparities divide.
